# Diagnosing lanolin contact allergy with lanolin alcohol and Amerchol L101

**DOI:** 10.1111/cod.13210

**Published:** 2019-03-19

**Authors:** Jannet Knijp, Derk P. Bruynzeel, Thomas Rustemeyer

**Affiliations:** ^1^ Department of Dermatology Amsterdam University Medical Centre Amsterdam The Netherlands

**Keywords:** Amerchol L‐101, cetyl alcohol, contact allergy, lanolin alcohol, patch testing, vehicle, wool alcohols

## Abstract

**Background:**

The prevalence of lanolin contact allergy in dermatitis patients varies from 1.2% to 6.9%. Different lanolin derivatives are used in patch testing.

**Objectives:**

To determine which combination of lanolin derivatives is most effective in patch testing for the diagnosis of lanolin contact allergy.

**Methods:**

A retrospective analysis of patients patch tested between 2016 and 2017 was performed. Patients were eligible if they had been tested with lanolin alcohol 30% pet., Amerchol L101 50% pet., and a supplementary series containing other lanolin derivatives. Lanolin alcohol and Amerchol L101 were tested in duplicate.

**Results:**

Of 594 patients, 28.6% (95% confidence interval [CI]: 25.1%‐32.3%) had a positive patch test reaction to at least one lanolin derivative. Reactions to lanolin alcohol (14.7%, 95%CI: 11.3%‐18.2%) and Amerchol L101 (15.0%, 95%CI: 11.5%‐18.5%) were common in the routinely tested series. Reactions to other test preparations were significantly less frequent (*P* < 0.05). The addition of Amerchol L101 to lanolin alcohol significantly increased the number of positive cases (odds ratio 1.79, *P* < 0.001).

**Conclusions:**

The combination of lanolin alcohol and Amerchol L101 is effective in patch testing for the diagnosis of lanolin contact allergy. Routinely testing with other lanolin derivatives may not be worthwhile, as it detects only a few additional patients.

## 1 INTRODUCTION

Lanolin is a complex mixture of sterols (wool wax alcohols), fatty alcohols and fatty acids with a varying composition.^1^ It is derived from a secretion of the sebaceous glands of sheep.^1^, ^2^, ^3^ Because of its emollient properties, lanolin is used in cosmetic products and topical medicaments.^2^, ^4^ Allergic contact dermatitis caused by lanolin typically develops after repeated or prolonged topical exposure, especially on damaged skin.^5^ Atopic dermatitis, leg ulcers and lower‐extremity venous stasis dermatitis have been identified as risk factors for the development of lanolin contact allergy.^1^, ^2^, ^4^, ^6^, ^7^, ^8^ The reported prevalence of lanolin sensitization in referred dermatitis patients has varied from 1.2% to 6.9% between several studies.^4^, ^6^, ^7^, ^9^, ^10^ Lanolin alcohol 30% pet. is the standard patch test agent for diagnosing lanolin contact allergy, and has been included in the European baseline series (EBS) since 1969.^1^, ^2^, ^6^, ^11^ However, supplementary patch testing with other lanolin derivatives seems to improve the identification of lanolin‐sensitive patients.^2^, ^10^, ^12^ Particular attention is being payed to Amerchol L101 50% pet., which is a mixture of 10% lanolin alcohols and mineral oil.^7^, ^11^ Several studies reported more reactions to Amerchol L101 than to lanolin alcohol.^2^, ^10^, ^11^, ^13^ Moreover, in a recent multicentre study,^13^ a group of 79 969 patients were simultaneously tested with both lanolin alcohol and Amerchol L101: more patients reacted only to Amerchol L101 (2.05%) than only to lanolin alcohol (1.19%) (*P* < 0.001). This raises the question of which lanolin products (eg, acetylated lanolin, hydrogenated lanolin, or ointments such as Eucerin) may also be used as patch test preparations. The frequencies of reactions to different lanolin derivatives have been studied, but there is limited information on the quality of these test preparations. The clinical relevance—the responsibility of the putative allergen for the (current or past) dermatitis—and reliability are useful in assessing this quality. The reliability can be assessed by means of the reaction index (RI), introduced by Brasch in 1992, ranging from −1 to 1.^14^ He proposed that an ideal patch test should have an optimal discriminatory power. A patch test is acceptable when the RI is >0, with a higher number of positive reactions than irritant and doubtful reactions.^14^, ^15^

The primary aim of this study was to determine which combination of lanolin derivatives is most effective in patch testing for the diagnosis of lanolin contact allergy. Therefore, we investigated the reaction prevalences, reliability and clinical relevance of individual lanolin derivatives. Moreover, we analysed the additional value of supplementary testing with lanolin alcohol.

## 2 MATERIALS AND METHODS

### 2.1 Patients

A retrospective analysis was performed on data of 594 patients who were patch tested in the VU University Medical Centre (VUmc) between January 1, 2016 and December 31, 2017. Patients were selected if they were routinely tested with the EBS, containing lanolin alcohol, and the routine supplementary series, containing Amerchol L101 (n = 594), and if they were additionally tested with our wool alcohol series, containing seven lanolin preparations, and/or with our topical medicament series, containing six lanolin preparations (Tables 1 and 2). These series include both lanolin‐containing ointments and components of lanolin. The patch test results obtained with this combination of preparations make it easier to advise the patient which products to avoid. The series were simultaneously tested in all patients, except for 41.

**Table 1:** Patch test reactions to lanolin derivatives (n = 594)

Patch test preparation	Total tested	Questionable reactions^a^	Positive reactions	Reaction prevalence, % (95%CI)
Routinely tested series				
European baseline series:				
Lanolin alcohol (30% pet.)	594	41	52	8.8 (6.5‐11.0)
Routine supplementary series:				
Amerchol L101 (50% pet.)	594	49	61	10.3 (7.8‐12.7)
Supplementary series				
Wool alcohol series				
Lanolin alcohol (30% pet.)	407	44	60	14.7 (11.3‐18.2)
Amerchol L101 (50% pet.)	407	49	61	15.0 (11.5‐18.5)
Cetearyl alcohol (20% pet.)	407	17	6	1.5 (0.3‐2.6)
Lanolin (adeps lanae) (30% pet.)	407	12	13	3.2 (1.5‐4.9)
Eucerin (“as is”)	407	31	10	2.5 (1.0‐4.0)
Unguentum lanette (“as is”)	407	22	10	2.5 (1.0‐4.0)
Cremor lanette (“as is”)	407	53	48	11.8 (8.7‐14.9)
Topical medicament series:				
Cetyl alcohol (20% pet.)	215	3	2	0.9 (0 to 2.2)
Stearyl alcohol (20% pet.)	215	6	2	0.9 (0 to 2.2)
Cetearyl alcohol (20% pet.)	214	7	1	0.5 (0 to 1.4)
Eucerinum anhydricum (“as is”)	215	5	2	0.9 (0 to 2.2)
Cera cetomacrogolis (“as is”)	212	2	1	0.5 (0 to 1.4)
Cera lanette (“as is”)	212	8	10	4.7 (1.9 to 7.6)

Abbreviation: CI, confidence interval.

Lanolin alcohol, Amerchol L101, cetearyl alcohol and adeps lanae were obtained from AllergEAZE or Chemotechnique Diagnostics. Ointments in the wool alcohol series (Eucerin, unguentum lanette, and cremor lanette) were produced by Fagron. Preparations in the topical medicament series were produced by the VUmc pharmacy. Cera lanette was applied as a pellet, and the others were dispersed in pet.

a Doubtful and irritant reactions.

**Table 2:** Composition of the preparations

Ointment	Composition
Eucerin	Lanolin alcohols (6%), paraffin, sorbic acid, and water
Eucerinum anhydricum	Eucerin without water
Unguentum lanette	Cera lanette SX, pet., paraffin, and isopropyl myristate
Cremor lanette	Cera lanette SX, cetiol V, sorbitol, sorbic acid, and water
Cera lanette	Cetearyl alcohol (90%) and sodium lauryl sulfate (10%)
Cera cetomacrogolis	Cetearyl alcohol (80%) and cetomacrogol (20%)

### 2.2 Patch testing and data reports

Patch testing was performed with Van der Bend (Brielle, The Netherlands) square chambers on tape. Patch test preparations were obtained from Van der Bend (brands AllergEAZE and Chemotechnique Diagnostics, Vellinge, Sweden), Fagron (Capelle aan den Ijssel, The Netherlands), and the local pharmacy of the VUmc (Amsterdam, The Netherlands). Patch test readings were performed on day (D) 2, D3 or D4 and, if required, on D6 or D7. Reactions were scored according to the recommendations of the ICDRG and the ESCD.^16^, ^17^ Patient records were abstracted from the European Surveillance System of Contact Allergies^18^ database of the VUmc. Positive reactivity to a test preparation was reported if there was a positive reaction (+, ++, or +++) on D3 or at a later reading time.^17^ Irritant reactions and doubtful (?+) reactions were combined and reported as “questionable” reactions, as it can be difficult to distinguish between these reactions.^6^, ^17^ The RI was calculated as (*p* – *q*)/(*p* + *q*), with *p* being the number of positive reactions to a patch test, and *q* being the number of questionable reactions to a patch test. The additional value of supplementary testing was calculated as the difference between reaction frequencies. The decision on relevance was based on criteria as proposed by Johansen et al^16^ and Lachapelle et al.^17^ Positive reactions with known clinical relevance were assessed as: “certain” when the clinician was convinced that the allergen was causative for the dermatitis; “probable” when there was a strong relationship between the allergen and dermatitis; “possible” when the relationship between the allergen and dermatitis was less clear, but the allergen was nevertheless suspected to have caused the dermatitis; and “unlikely/not” when the allergen was not suspected.^8^ If the relevance could not be established, it was recorded as “unknown”.^16^, ^17^ In this study, “certain” and “probable” scores were combined to reflect the highest relevance scores.

### 2.3 Statistical analysis

All patient characteristics along with the tested series were included as variables in a multivariate logistic regression analysis. Backward elimination with a significance level of *P* < 0.05 was used to select the best set of risk factors. The interaction between predictors of lanolin contact allergy was evaluated with Spearman's rho correlation test. Differences in reaction frequencies were evaluated with the one‐sized *z*‐test for proportions. The McNemar test for paired data was used to evaluate the additional value of supplementary testing. Statistical analysis was performed with spss. A *P* value of <0.05 was considered to be statistically significant.

## 3 RESULTS

In the study population of 594 patients, 170 patients (28.6%, 95% confidence interval [CI]: 25.1%‐32.3%) had a positive patch test reaction to at least one lanolin derivative and were defined as allergic to lanolin. Patients with atopic dermatitis were more likely to have lanolin contact allergy (*P* = 0.008, odds ratio [OR] 1.75, 95%CI: 1.16‐2.63), and patients with aged ≥40 years were less likely to have lanolin contact allergy (*P* = 0.007, OR 0.57, 95%CI: 0.38‐0.86). Moreover, the supplementary tested series were significantly associated with lanolin contact allergy (*P* = 0.002) (Table 3). There were correlations between atopic dermatitis and an age of <40 years (*P* < 0.001, OR 4.3, 95%CI: 3.05‐6.17) and between atopic dermatitis and the supplementary tested series (*P* < 0.001).

**Table 3:** Patient characteristics (n = 594)

		Total n = 594	Lanolin‐allergic n = 170	Lanolin‐negative n = 424
Age (y)	Mean (SD)	39.9 (17.62)	35.7 (17.00)	41.6 (17.61)
Age group, no.(*P* = 0.007)	Median (range)	40.0 (4–89)	33.5 (4–89)	42.5 (6–89)
	<40 y	290	106	184
	≥40 y	304	64	240
Sex, no.	Females	404	115	289
	Males	190	55	135
Atopic dermatitis, no. (*P* = 0.008)	Yes	301	110	191
	No	268	54	214
	Unknown	25	6	19
Tested series^a^ (*P* = 0.002)	Wool alcohol series	379	128	251
	Topical medicament series	187	32	155
	Both series	28	10	18

Abbreviation: SD, standard deviation.

Only *p*‐values statistically significant are presented.

a Patients were always tested with the European baseline series and the routine supplementary series containing lanolin alcohol and Amerchol L101.

The reaction frequency for Amerchol L101 in the wool alcohol series (15.0%, 95%CI: 11.5%‐18.5%) was significantly higher than the reaction frequencies for other test preparations (*P* < 0.05), except for lanolin alcohol in the wool alcohol series (14.7%, 95%CI: 11.3%‐18.2%) (*P* = 0.47). In the routinely tested series, the reaction frequencies for lanolin alcohol (8.8%, 95%CI: 6.5%‐11.0%) and Amerchol L101 (10.3%, 95%CI: 7.8%‐12.7%) were comparable (*P* = 0.38) (Table 1). Of all positive reactions, only five were scored as ++ reactions; all other reactions were scored as +.

**Figure 1:**
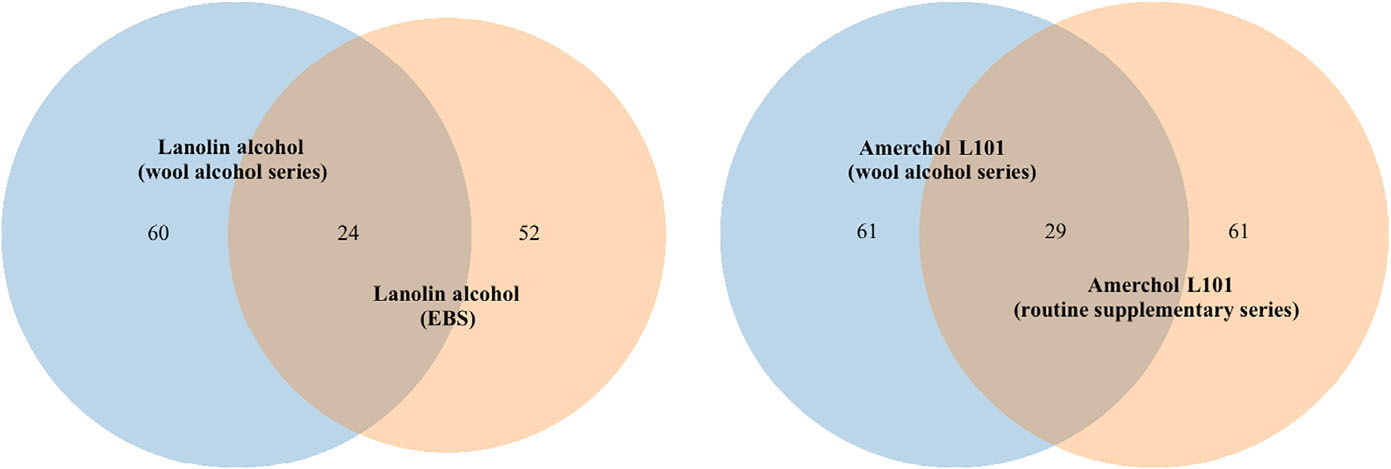
Venn diagram of lanolin alcohol and Amerchol L101 tested in duplicate. Shown are the numbers of positive reactions to the test substance in the wool alcohol series and the routinely tested series: the European baseline series (EBS) and routine supplementary series. The reproducibility was 27.3% and 31.2%, respectively

Both lanolin alcohol and Amerchol L101 were tested in duplicate, and showed discordant reactivity. Regarding lanolin alcohol, 88 patients had positive reactions in one or in both series, but only 24 of these had positive reactions in both series. Regarding Amerchol L101, 93 patients had positive reactions in one or in both series, but only 29 of these had positive reactions in both series. Thus, the reproducibility of positive reactions was 27.3% (24/88) for lanolin alcohol and 31.2% (29/93) for Amerchol L101 (Figure 1). Only 9 of the 60 (15%) positive reactions to lanolin alcohol in the wool alcohol series coincided with questionable reactions to the analogous agent in the routinely tested series. For Amerchol L101, only 5 of the 61 (8.2%) positive reactions in the wool alcohol series coincided with questionable reactions in the routinely tested series.

The RI was calculated for lanolin alcohol in the routinely tested series (RI: 0.12) and in the wool alcohol series (RI: 0.15), for Amerchol L101 in the routinely tested series (RI: 0.11) and in the wool alcohol series (RI: 0.11), and for cremor lanette (RI: −0.05). For other preparations, no RI was calculated, as this index is only useful with high numbers of positive reactions.^14^ Of all positive reactions, 69.0% were of current relevance, and 3.8% were of past relevance; for 27.1%, the relevance was unknown. However, Amerchol L101 in the routinely tested series had a high proportion of “unknown” scores (67.2%). On the basis of the reactions with known relevance (excluding “unknown” scores), the proportions of the combined “certain” and “probable” relevance scores ranged from 70.0% for lanolin alcohol and Amerchol L101 in the routinely tested series to 85.0% for cremor lanette, only including the preparations with ≥20 positive reactions.

The addition of Amerchol L101 to lanolin alcohol in the routinely tested series gave 35 (87 – 52) additional positive cases as compared with testing with lanolin alcohol only (*P* < 0.001, OR 1.79, 95%CI: 1.24‐2.58). The addition of the supplementary series to the routinely tested series accounted for 83 (170 – 87) extra diagnoses of lanolin contact allergy, which comprised 48.8% of the overall number of diagnoses (83/170) (*P* < 0.001, OR 2.34, 95%CI: 1.75‐3.12). Especially in patients additionally tested with the wool alcohol series only, the additional value of supplementary testing was considerable, with 71 (128 – 57) extra diagnoses (*P* < 0.001, OR 2.88, 95%CI: 2.02‐4.10). After exclusion of the patients with a different test date for the routinely tested series than for the supplementary series (n = 41), the study population contained 553 patients. Statistical analysis of this population did not yield different results regarding the additional value of supplementary testing or the discordance in positivity of the allergens tested in duplicate.

## 4 DISCUSSION

### 4.1 Lanolin alcohol and Amerchol L101

We confirmed that adding Amerchol L101 to lanolin alcohol in routine patch testing has additional diagnostic value in detecting lanolin contact allergy.^2^, ^10^, ^11^, ^13^ The highest prevalence of positive reactions was observed for both lanolin alcohol and Amerchol L101. Moreover, when the reliability was assessed by means of the RI, the results indicated lanolin alcohol and Amerchol L101 to be acceptable patch test preparations in both the routinely tested series and the supplementary series.^14^ It has been suggested that Amerchol L101 has irritant properties attributable to the mineral oil that it contains, resulting in false‐positive reactions.^6^, ^19^ Furthermore, mineral oil could function as a penetration enhancer. The slightly higher reaction frequencies for Amerchol L101 than for lanolin alcohol may be attributable to these properties. In our study, interpretation of positive reactions to Amerchol L101 in the routinely tested series was conservative: the clinical relevance of 67.2% of the reactions was unknown. We cannot exclude the possibility that some reactions were false‐positives attributable to irritation.

Brasch et al^20^ investigated reproducibility with concomitant patch testing of allergens on both sides of the back. The reproducibility for lanolin alcohol was 55%.^20^ In our study, the reproducibility of positive reactions to lanolin alcohol and Amerchol L101 was 30%. Moreover, in only a few cases did a positive reaction in one series coincide with a questionable reaction to the analogous agent in the other series. We have to keep in mind that low reproducibility is not uncommon in patch testing. It has been described with several allergens other than lanolin, varying from 36.0% to 53.9%.^2^, ^21^ The irritancy of the test preparations and the individual susceptibility may play a role in the low reproducibility. Theoretically, this could imply that only patients with positive reactions to both preparations have to be regarded as allergic to lanolin. However, this does not necessarily mean that a patient with only one positive reaction is not allergic to lanolin. Therefore, the diagnosis must be based on the history of the patient in combination with the test results. Importantly, low reproducibility implies that a patch test can give a negative result in a lanolin‐sensitive patient. This indicates that repeated patch testing may be needed when the suspicion of lanolin contact allergy is high.

### 4.2 Supplementary testing

Although the additional diagnostic value of the supplementary series was considerable, we have doubts about the usefulness of all patch test preparations in these series. In the wool alcohol series, the discordant reactivity to lanolin alcohol and Amerchol L101 as compared with the results with the routinely tested series mostly contributed to additional positive cases. Furthermore, cremor lanette had a high reaction prevalence (11.8%, 95%CI: 8.7%‐14.9%). However, on the basis of our findings, the usefulness of screening with cremor lanette seems to be limited. As cremor lanette is a mixture of different ingredients (cetearyl alcohol, sodium lauryl sulfate, cetiol V, sorbitol, sorbic acid, and water), positive reactions to this preparation may be attributable to sensitivity to one of the additives. In addition, the RI of <0 indicates that this preparation might be unreliable for patch testing. In the topical medicament series, the test preparations had relatively low reaction prevalences (<1.0%), except for cera lanette (4.7%, 95%CI: 1.9%‐7.6%). However, the quality of cera lanette as a patch test preparation is debatable, as this solid material was tested as a pellet, possibly producing false‐positive reactions resulting from pressure. Besides that, it contains the emulsifier sodium lauryl sulfate, which has irritant properties. In addition to these considerations, a reaction to a test preparation could also be caused by impurities.^6^ Consequently, the topical medicament series is not very effective for detecting additional patients with lanolin contact allergy, but can be used when constituents of topical medicaments and cosmetics, in particular, are suspected of causing dermatitis. Of note, it is important to test such patients with their own products and topical medicaments.

### 4.3 Prevalence and risk factors

Our study population consisted of patients for whom there was a high suspicion of lanolin contact allergy, as they were selected to be additionally tested with lanolin derivatives. Moreover, the patients were referred for tertiary care. As a result, the prevalence of patients with lanolin contact allergy (28.6%, 95%CI: 25.1%‐32.3%) is higher than is normally reported.^4^, ^6^, ^7^, ^9^, ^10^ The positive association between atopic dermatitis and lanolin contact allergy has previously been reported.^4^, ^7^, ^8^ An impaired skin barrier in atopic patients, together with the prolonged use of lanolin‐containing topical medicaments, possibly leads to an increased risk of sensitization. On the other hand, the association may be explained by false‐positive reactions attributable to irritation, which is more often seen in atopic skin.^5^, ^8^ The association between an age of <40 years and lanolin contact allergy was not in line with the literature, as previous studies reported a positive association between higher age and lanolin contact allergy.^6^, ^7^ The location of dermatitis may play a part in the association between age and lanolin contact allergy, for example, leg ulcers in the elderly. We did not include the primary site of dermatitis in our study, as other studies have, which might be the reason for diverging results. However, the patient population with leg ulcers referred to our clinic has, in practice, not been treated with lanolin‐containing ointments for many years, owing to the use of modern wound dressings. As a consequence, the number of patients with leg ulcers is low in our clinic.

### 4.4 Future perspectives

Our results showed higher numbers of questionable reactions than of positive reactions to 9 of 15 patch test preparations. This clearly suggests that the patch test concentration and/or the vehicle of some lanolin derivatives is not perfect. Further research, with patients with known positive or questionable reactions, is necessary to optimize patch test concentrations and the vehicle of lanolin derivatives. In addition, the relevance of a positive patch test reaction to lanolin can be difficult to establish. A use test, such as the repeated open application test (ROAT), is useful to assess the relevance of exposure to an allergen identified by patch testing.^22^ Further research comparing the results of a ROAT with positive patch test reactions to lanolin may clarify the results of patch testing with lanolin derivatives.

### 4.5 Strengths and limitations

A strength of our study is that we investigated which lanolin derivatives contributed to the additional diagnostic value and that we assessed the quality of the test preparations in this regard.^10^, ^12^ Moreover, all patients were patch tested in the same clinic and under the same circumstances, resulting in an equal set of outcomes. Our study also has limitations. First, the preparations used for patch testing were obtained from different producers, and might vary in their composition. Second, a bias could have been introduced by the fact that relatively few children and leg ulcer patients were included in our study population. This makes age as a potential risk factor difficult to interpret. Finally, we did not include the strength of the positive reaction (+, ++, or +++) in the analysis, because almost all positive reactions were + reactions.

## 5 CONCLUSION

Lanolin contact allergy is frequently seen in referred dermatitis patients. It is a clinical problem, especially for those using lanolin‐containing topical medicaments. The diagnostic test preparations might be improved. Our study has shown that lanolin alcohol and Amerchol L101 constitute an effective combination of patch test preparations for diagnosing lanolin contact allergy. In cases with a high suspicion of lanolin contact allergy, but negative patch test results, it may be necessary to repeat patch testing with these preparations. As other lanolin derivatives contributed to only a few additional positive cases, it may not be worthwhile using them routinely for diagnosing lanolin contact allergy, but only in selected patients. Further research is necessary to improve the patch test concentration of lanolin derivatives and better define the clinical relevance of positive patch test reactions.

## CONFLICTS OF INTEREST

The authors have no conflicts of interest to report.
